# Empowering Community Health Workers With Scripted Medicine: Design Science Research Study

**DOI:** 10.2196/57545

**Published:** 2025-04-23

**Authors:** Dario Staehelin, Damaris Schmid, Felix Gerber, Mateusz Dolata, Gerhard Schwabe

**Affiliations:** 1 Department for Information and Process Management Eastern Switzerland University of Applied Sciences St. Gallen Switzerland; 2 Department of Informatics University of Zurich Zurich Switzerland; 3 Division of Clinical Epidemiology Department of Clinical Research University Hospital Basel Basel Switzerland; 4 Swiss Tropical and Public Health Institute University of Basel Basel Switzerland; 5 Swiss Tropical and Public Health Institute Allschwil Switzerland; 6 Department of Political and Social Sciences Zeppelin University Friedrichshafen Germany

**Keywords:** community-based health care, community health workers, collaboration engineering, algorithmic management, Scripted Medicine, task shifting, empowerment, mobile health, mHealth, digital health

## Abstract

**Background:**

The World Health Organization anticipates a shortage of 14 million health workers by 2030, particularly affecting the Global South. Community health workers (CHWs) may mitigate the shortages of professional health care workers. Recent studies have explored the feasibility and effectiveness of shifting noncommunicable disease (NCD) services to CHWs. Challenges, such as high attrition rates and variable performance, persist due to inadequate organizational support and could hamper such efforts. Research on employee empowerment highlights how organizational structures affect employees’ perception of empowerment and retention.

**Objective:**

This study aims to develop Scripted Medicine to empower CHWs to accept broader responsibilities in NCD care. It aims to convey relevant medical and counseling knowledge through medical algorithms and ThinkLets (ie, social scripts). Collaboration engineering research offers insights that could help address the structural issues in community-based health care and facilitate task shifting.

**Methods:**

This study followed a design science research approach to implement a mobile health–supported, community-based intervention in 2 districts of Lesotho. We first developed the medical algorithms and ThinkLets based on insights from collaboration engineering and algorithmic management literature. We then evaluated the designed approach in a field study in the ComBaCaL (Community Based Chronic Disease Care Lesotho) project. The field study included 10 newly recruited CHWs and spanned over 2 weeks of training and 12 weeks of field experience. Following an abductive approach, we analyzed surveys, interviews, and observations to study how Scripted Medicine empowers CHWs to accept broader responsibilities in NCD care.

**Results:**

Scripted Medicine successfully conveyed the required medical and counseling knowledge through medical algorithms and ThinkLets. We found that medical algorithms predominantly influenced CHWs’ perception of structural empowerment, while ThinkLets affected their psychological empowerment. The different perceptions between the groups of CHWs from the 2 districts highlighted the importance of considering the cultural and economic context.

**Conclusions:**

We propose Scripted Medicine as a novel approach to CHW empowerment inspired by collaboration engineering and algorithmic management. Scripted Medicine broadens the perspective on mobile health–supported, community-based health care. It emphasizes the need to *script* not only essential medical knowledge but also *script* counseling expertise. These scripts allow CHWs to embed medical knowledge into the social interactions in community-based health care. Scripted Medicine empowers CHW to accept broader responsibilities to address the imminent shortage of medical professionals in the Global South.

## Introduction

### Background

The World Health Organization projects a health worker shortage of 14 million by 2030 [1]. Many countries struggle to train and retain enough medical professionals [1], with the Global South disproportionately affected by this shortage [2]. In response to this situation, community health workers (CHWs) have been integral in alleviating the consequences of staff shortages and extending the health systems in many countries [3]. CHWs are laypeople residing within their communities who receive basic medical training to provide essential health services in their communities and to link patients to professional facility-based health care [3]. Whereas physicians are often rare, expensive, and concentrated in urban hubs, CHWs are available in vast numbers and comparably inexpensive. While CHWs have traditionally focused on maternal, neonatal, and infectious disease services, recent studies have investigated the feasibility of expanding CHWs’ responsibilities to tackle the rapidly growing noncommunicable disease (NCD) pandemic [4,5]. In task shifting, specific tasks are shifted from medical professionals to CHWs [6,7]. For example, many community-based health programs in the Global South shift screening and monitoring tasks for infectious diseases, such as HIV and tuberculosis, from medical professionals to CHWs. Task shifting may be a cost-effective approach to increasing the reach of health care delivery systems. It may reduce important access barriers by allowing for more decentralized health care delivery while freeing medical professionals’ resources [8].

Despite the increasing popularity of community-based health programs, attrition rates among CHWs are generally high, and studies often report high variability in CHW performance [9-13]. These persisting challenges are linked to inadequate organizational support [12-14], including insufficient training and economic opportunities [15,16] and a lack of appreciation and support from supervisors [9,14]. Further, CHWs reported other social challenges, such as a lack of support from their families and insufficient trust, linked to low uptake rates of services by the community [14].

### Empowering CHWs to Accept Broader Responsibilities

The empowerment literature reported high attrition rates and performance variability due to a lack of organizational support, especially in nurses [17-19]. Studies show that an organization needs to transfer power from supervisors to employees to create ideal work conditions. This transfer of power is called structural empowerment [20,21]. It emphasizes the necessity of providing employees with a work environment that enables them to carry out their work. Structural empowerment is achieved by providing employees *access to information*, *resources*, *support*, and *opportunities* [21,22]. The act of empowerment positively influences the perception of empowerment. Psychological empowerment builds on the idea that perceived control increases intrinsic motivation. It draws on prior research on self-efficacy [23] and self-determination [24] and is measured through the perception of *meaning*, c*ompetence, autonomy,* and *impact* [25]. Empowering employees increases retention rates and job satisfaction and decreases job strain [18,22,26]. Employees feel disempowered when lacking access to critical information (eg, through training), resources (eg, adequate pay), or support from peers and supervisors. Disempowered employees are less productive and more likely to change jobs.

Unsurprisingly, CHW empowerment is a growing topic of interest. However, CHW empowerment is a poorly defined concept despite its recent popularity. Researchers often use empowerment as a synonym for facilitating or enabling CHWs to carry out specific tasks without diving into the depth of its meaning. For example, CHWs are empowered to treat diabetes [27] or provide counseling for family planning [28]. Consequently, they often miss addressing the persisting challenges of inadequate organizational support that lead to high attrition rates and performance variability. Only a few articles introduce CHW empowerment conceptualizations that consider the sociotechnical aspects of community-based health care. They highlight how the lack of organizational support disempowers CHWs with social, economic, and cultural consequences [29-32].

### Mobile Health for CHWs

Mobile technologies often play a central role in efforts to empower CHWs in their work [33-35]. Recent studies demonstrate the potential of mobile health (mHealth) to address the challenges in CHW retention and performance [36], for example, through decision support [11,37-39]. Clinical decision support systems provide CHWs with the necessary clinical knowledge through medical algorithms to ensure the proper implementation of guidelines [37,40-42]. These explorations in empowering CHWs with decision-support tools align well with the premise of algorithmic management. Algorithmic management is a growing research topic in human-computer interaction and information systems research. It argues that management becomes a process embedded in technology rather than a human routine [43,44]. Studies have shown how algorithmic management can increase productivity without increasing job strain by managing the user’s work process [45,46].

The 5RSM framework describes 7 forms of algorithmic management for process restriction: *algorithmic* *recommending, restricting, requiring, rating, rewarding, sanctioning,* and *monitoring* [47]. Table 1 provides the original definition for each form of algorithmic management from Hirsch et al [47]. We complement the table with CHW-related examples.

**Table 1:** Forms and definitions of algorithmic management [47], with community health toolkit (CHT)–related examples.

Form	Definition (how the algorithm aligns worker behavior with organizational goals)	Example (CHT application)
Recommending	The algorithm automatically suggests specific behavior to the worker that takes the form of either explicit recommendations for action or implicit forms (such as nudges).	Medical algorithms propose a task prioritization that CHWs^a^ can adopt to organize their workflow.
Restricting	The algorithm withholds information or limits the scope of action for the worker.	Medical algorithms hide recommendations for a drug prescription in case of reported intolerance.
Requiring	The algorithm instructs workers to perform specific actions that they cannot reject without negative consequences.	Medical algorithms require CHWs to perform tasks by a specific due date.
Rating	The algorithm ranks workers or allows third parties to rate work behavior and outcome.	Medical algorithms display leader boards comparing the number of clients CHWs have counseled.
Rewarding	The algorithm assigns rewards for work behaviors that align with organizational goals.	Medical algorithms issue nonmonetary (eg, digital badges) and monetary rewards (eg, performance-based compensation).
Sanctioning	The algorithm issues penalties for work behaviors that do not align with the organizational goals.	Medical algorithms reduce performance-based compensation if tasks are not completed in due time.
Monitoring	The algorithm enables supervision of the work process by the worker or a third party by collecting, aggregating, and displaying data on working behavior.	Medical algorithms provide performance dashboards with performance indicators such as the number of delayed task completions per month.

^a^CHW: community health workers.

While algorithmic management offers valuable tools for empowering CHW with mHealth, we believe 2 dimensions require specific consideration for community-based health care. *Recommending* might only be applied in areas where patient safety is not at stake. For example, algorithms must *require* instead of *recommending* CHWs to perform specific tasks such as distributing medication on time to ensure patient safety. Work termination, the strongest form of *sanctioning*, should not be performed by algorithms. Work termination has far-reaching consequences as communities would temporarily be left without their CHW.

Digital CHW empowerment is associated with the improvement of patient knowledge and attitude [48], disease surveillance and health coverage [33], and identification of health priorities in the communities [49]. These efforts often aim to address the root causes of CHW disempowerment [35]: the lack of organizational support and appreciation (eg, by supervisors) [9,14]; inadequate training and learning opportunities [15]; and insufficient monetary compensation [16].

However, the growing CHW empowerment literature mentioned earlier criticizes the unilateral focus on clinical knowledge to capacitate CHWs [29,30,50]. Research often abstracts from the context in which the clinical knowledge is applied [51]. It studies how technology *empowers* CHWs to become better and more reliable at diagnosing and monitoring diseases in the field. Consequently, the importance of a functioning CHW-client interaction is often omitted [52,53]. A recent study highlights the persisting, often implicit, view of CHWs as sensors for the health system because of this unilateral consideration [54]. CHWs are often deployed to *sense* the health status of their community by diagnosing and monitoring specific diseases (eg, HIV). However, they are not autonomous actors who offer treatment services. This perception stands against the proclaimed active role CHWs should claim to address predicted staff shortages [4,50]. Consequently, research lacks a comprehensive approach that embeds clinical knowledge in its application context to empower CHWs to accept broader responsibilities.

### Collaboration Engineering

Current research often abstracts from the social interactions—the collaboration between CHWs and patients—in community-based health care. However, we argue that community-based health care is not the outcome of an individual’s effort (ie, CHW) or technology. It is a collaborative process between CHWs, their clients, and the health system. Seen as a collaborative process, community-based health care would likely benefit from insights from collaboration engineering research. Collaboration engineering originates from research on group support systems (GSSs) [55]. This research studies how technology enhances group collaboration by reducing process losses, such as conformity pressure or the conversation domination of 1 or a few collaborators [56]. Despite the substantial benefits, GSS adoption struggled as they require professional facilitators who design and conduct GSS-supported group collaborations [56,57]. However, facilitators are a rare and expensive resource, requiring extensive training and experience [58].

The premise of collaboration engineering is to drastically reduce costs and training efforts by enabling virtually anyone to design and conduct group collaboration. Briggs et al [59] described this as the “facilitator in a box.” The core assumption is that high value and recurring tasks can be predesigned to empower nonexperts through process restrictiveness [57,60]. This restriction quickly allows nonexperts to carry out expert tasks without extensive training and experience. However, collaboration engineering stresses that they must benefit users and offer adequate flexibility to avoid adverse effects [59,60]. The literature describes enabling nonexperts to fruitfully collaborate by restricting them in the process through (1) a carefully designed series of activities, (2) the configuration of technology to support behaviors within each activity, and (3) guidance on the behaviors (including constraints) they should perform in each activity [59]. Collaboration engineering research proposes ThinkLets to guide the behaviors in these activities. ThinkLets are tools to “codify expertise such that it would be easy for [nonexperts] to learn and reuse.” Similar to a cooking recipe, they consist of explicit instructions on collaboration behaviors, including instructions on configuring and using support tools [57]. ThinkLets formalize the required expertise for nonexperts to reach predictable and repeatable outcomes, increasing collaboration performance and outcomes [61].

On the basis of these insights, we argue that collaboration engineering is a promising approach for CHW empowerment as its underlying premise closely resembles global struggles in health care. The fundamental problem that collaboration engineering aims to resolve is that of scarce, valuable resources. Facilitators are replaced with inexpensive and plentiful nonexperts, who receive tools to facilitate collaboration in a specific context successfully. Collaboration engineering breaks processes into repeatable and formalized pieces, preconfigured technology, and guidance on required behaviors. From this perspective, collaboration engineering and community-based health care adopt a similar approach. Medical professionals facilitate their medical consultations based on their formal training and a repertoire of scripts grown by experience. This training is time-consuming and expensive. Likewise, CHWs are the nonexpert collaborators in medical consultations. They are available in large numbers and at comparably low costs. They lack critical knowledge and expertise that must be compensated through process restrictions. Thus, medical professionals are trained facilitators, and CHWs are nonexperts facilitating health care within a limited domain.

Following this analogy, we claim collaboration engineering offers efficient and effective task shifting tools that “standardize the performance and interpretation of certain tasks” [6]. Consequently, CHWs could be empowered to accept broader responsibilities and perform tasks beyond diagnosing and monitoring patients. In turn, empowered CHWs might be retained longer, reducing attrition rates. Despite this potential value, there is a lack of research studying how collaboration engineering can formalize crucial knowledge to facilitate task shifting from medical professionals to CHWs.

### Research Objective

This study addresses this research gap by developing Scripted Medicine, an approach for CHW empowerment inspired by research on collaboration engineering and algorithmic management. The insights from algorithmic management align well with restricting collaboration processes through (1) a carefully designed series of activities and (2) the configuration of technology to support behaviors within each activity. In community-based health care, mHealth combines both principles as medical algorithms represent the series of activities and provide the user with the functionalities required to perform these activities. Collaboration engineering offers ThinkLets as a tool to guide CHWs’ behaviors throughout the activities. They convey expert knowledge in predictable and repeatable recipe-like scripts that make the knowledge easily accessible for nonexperts. Accordingly, we formulate the design goal and solution objectives demonstrated in Textbox 1.

**Design goal and solution objectives.**

**Design goal**

- Empower community health workers with Scripted Medicine to accept broader responsibilities in noncommunicable disease care

**Solution objectives**

- “Medical Knowledge”
  Convey medical knowledge through a carefully designed series of activities and preconfigured technology by drawing on insights from algorithmic management
- “Counseling Knowledge”
  Convey knowledge through guidance on specific behaviors in the activities in ThinkLets

## Methods

### Overview

We followed the design science research methodology proposed by Peffers et al [62] to develop the Scripted Medicine approach for CHW empowerment. Design science research is especially suitable as it aims to create artifacts that address a practical problem [63]. Such artifacts include software designs, theoretical models, or approaches [62]. While originating from information systems and computer science, design science research is increasingly applied in medical informatics to study emerging phenomena and generate new knowledge through a design approach [64-68]. The following subsections introduce the ComBaCaL (Community Based Chronic Disease Care Lesotho) project and describe our implementation of the design science research methodology. This manuscript focuses on the diabetes pilot of the ComBaCaL project.

### Ethical Considerations

The pilot is approved by the Ministry of Health Research and Ethics Committee Lesotho (ID 176-2021). All participants gave verbal informed consent to participate in the study. The obtained data was de-identified. The participants did not receive any compensation for participating in the study.

### ComBaCaL: Community-Based NCD Care

ComBaCaL is an innovative initiative to address the escalating NCD epidemic in Lesotho. The 5-year project used a community-based model, leveraging CHWs and mHealth to enhance NCD prevention, screening, diagnosis, and care.

One key aspect of ComBaCaL is its emphasis on empowering CHWs to play a pivotal role in delivering NCD care at the community level. Lesotho successfully decreased HIV transmission and AIDS-related deaths mainly due to the broad rollout of HIV testing and antiretroviral treatment. Previous studies explored the potential of decentralized, home-based service delivery for HIV care in the setting [69-71]. The project aimed to continue expanding the responsibilities of CHWs horizontally (ie, other diseases) and vertically (ie, offer treatment).

ComBaCaL is among the first projects to expand CHWs’ responsibilities to provide treatment for NCDs through task shifting. The project strategically focuses on 2 critical NCDs: type 2 diabetes and hypertension, with a comprehensive care package beyond screening and monitoring. ComBaCaL follows a more comprehensive approach to community-based care that includes lifestyle counseling and prescription of first-line treatments for uncomplicated type 2 diabetes and hypertension. The goal was to provide accessible NCD care within the community, thus minimizing the need for travel to the often distant health care facilities without compromising patient safety and care quality.

To achieve this goal, ComBaCaL used mHealth, specifically a community health toolkit (CHT), to capacitate CHWs. These tools guide CHWs through clinical algorithms on tablets, enabling them to effectively screen and monitor NCDs, prescribe first-line treatments, and provide necessary care within the community.

The CHT is an open-source toolkit for community-based health care that reflects the needs of the local context [72]: a community-based perspective, an offline-first approach, and flexibility in configuring medical algorithms. These tools should streamline CHW activities and facilitate decision support based on entered data, ensuring effective and accurate care provision.

ComBaCaL is a multidisciplinary implementation research project that aims to improve care for chronic diseases in rural Lesotho by developing and implementing innovative community-based health service delivery models. The project consortium includes SolidarMed, a local nonprofit organization; the Division of Clinical Epidemiology at the University Hospital Basel; the National University of Lesotho; the Lesotho Ministry of Health; and the Department of Informatics at the University of Zurich.

### Design and Development

An interdisciplinary and multicultural project team adopted a human-centered approach to gather extensive requirements for the envisioned community-based health program with support from students in Lesotho and Switzerland [73,74]. This approach allowed the project team to include all relevant stakeholders and their needs. The program was developed in 2 streams between February 2021 and April 2022. In the first stream, the project team developed medical algorithms instantiated in the CHT, named the ComBaCaL app. In collaboration with medical experts, informatics students from a university in Switzerland build medical algorithms that contain step-by-step guidance in registering, screening, diagnosing, and (if necessary) treating clients for diabetes. The algorithms were developed using agile methods and tested before the intervention. In the second stream, a group of 4 researchers iteratively codeveloped ThinkLets. The researchers developed 9 ThinkLets covering the social interactions associated with the medical algorithm’s guidance. They especially considered the interrelation between medical procedures and social interactions between CHWs and their patients. The ThinkLets were revised and finalized after the CHW training to incorporate learning from practice sessions.

### Demonstration and Evaluation

The concept was evaluated with 10 newly recruited CHWs in 2 districts in Lesotho in Spring 2022. The communities elected the CHWs according to the Lesotho Village Health Policy. The scope of the evaluation covers 2 weeks of training and approximately 12 weeks of field experience, during which CHWs provided diabetes counseling in their community.

The first week of training focused on clinical knowledge. Medical experts educated CHWs on diabetes risk factors, pathophysiology, diagnosis, and treatment. Practical sessions on medical procedures, such as capillary blood glucose measurement, complemented the lectures and allowed CHWs to practice with peers. The second training week focused on embedding the gained clinical knowledge into the interactions between CHWs and their clients. The practice-oriented counseling training was organized in modules along the counseling process. Each module was built on the previous one to cover the whole interaction when combined. The modules all followed the same approach. First, the trainers introduced the relevant ThinkLet to the plenum. They explained the aim, reason, and process of each ThinkLet and answered questions. A trainer demonstrated how a ThinkLet intends to support the CHWs in handling the medical algorithm on a tablet app and their interaction with clients. A total of 2 CHWs then applied the ThinkLet in a roleplay, with the remaining CHWs watching and asking questions. Second, the CHWs practiced on each other in groups of 5 by applying the clinical knowledge in the algorithms in its social context supported by ThinkLets. Third, the groups met again in the plenum to discuss what they had learned and to answer open questions. This approach was repeated until the CHWs counseled their peers, from welcoming to saying goodbye. The training concluded with 2 days in a nearby village where the CHWs applied their knowledge and skills in a real-world setting with people closely resembling their future clients.

### Data Collection and Analysis

#### Overview

**Figure 1:**
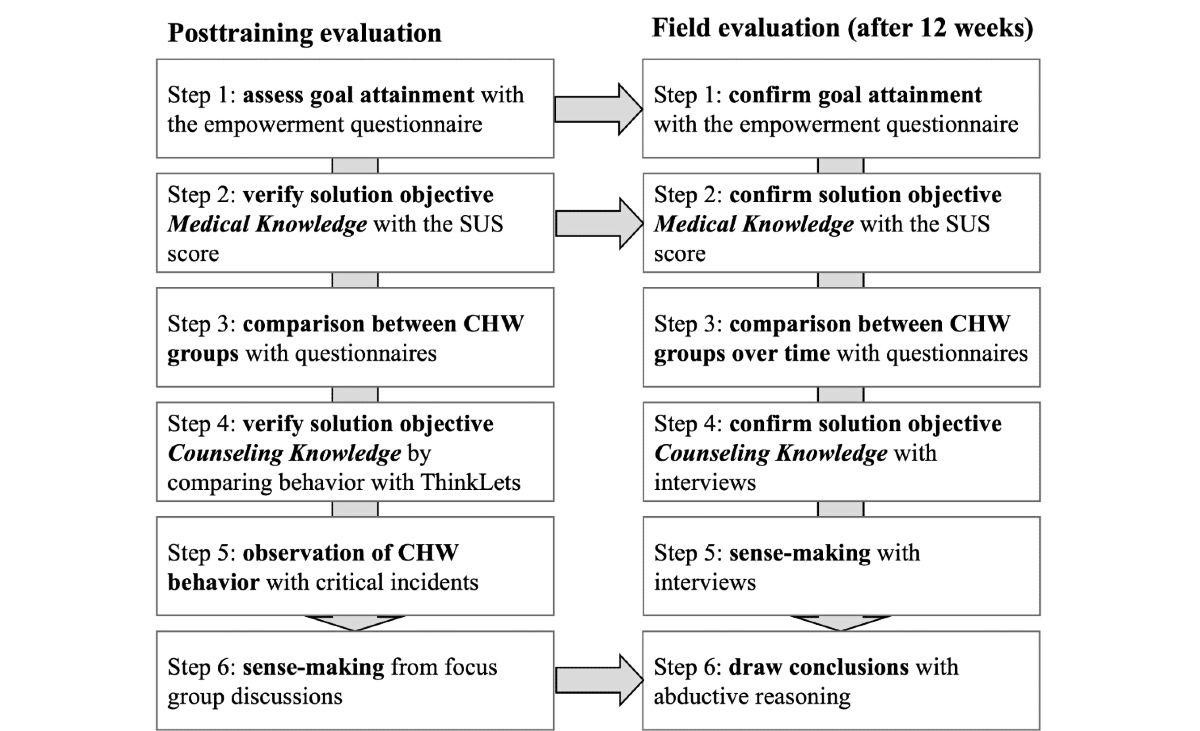
An abductive analysis approach for the posttraining and field evaluation. CHW: community health worker; SUS: System Usability Scale.

We followed an abductive approach to study how Scripted Medicine empowers CHWs to accept broader responsibilities in NCD care. Deductive and inductive approaches aim to test a theory objectively (ie, deductive) or generate a theory from interpretation (ie, inductive). Compared to these approaches, abductive research balances empirical data and existing theory [75]. This balance makes abductive reasoning suitable for design science research and this study as it considers a theory (ie, empowerment) and empirical data to generate new knowledge [76]. We conducted our abductive approach in 2 rounds, as depicted in Figure 1. Throughout the data collection, the CHWs always could speak Sesotho to express themselves in the best way possible. Native speaking Basotho either participated in the focus group discussion or conducted the interviews if preferred. Questionnaires were in Sesotho and English languages.

#### Posttraining Evaluation

We collected data during the CHWs’ training. We captured video recordings during the training sessions to study the CHWs’ learning curve. At the end of the training, the CHWs participated in a focus group discussion to reflect on their training experience and expectations toward their new role. We divided the CHWs into 2 groups of 5 to give each CHW enough space to speak. Finally, each CHW completed a questionnaire on structural and psychological empowerment [17,25] and rated the ComBaCaL app on the System Usability Scale (SUS) [77] (see Multimedia Appendix 1 [17] and Multimedia Appendix 2 [77] for the questionnaires).

We conducted the data analysis in 6 steps. First, we began our analysis by focusing on the questionnaire data to assess goal attainment regarding the CHWs’ perception of structural and psychological empowerment. We calculated the median and mean for each item and item group (where applicable). Second, we calculated the SUS score for the ComBaCaL app. The SUS evaluates the quality of the process restrictions instantiated in the ComBaCaL app through (1) the designed sequence of activities and (2) the configuration of the app, and, therefore, verifies solution objective 1. Third, we compared the results between the Mokhotlong and Butha Buthe groups (5 CHWs each). This step elicited interesting insights to guide the subsequent observation and interview data analysis. We then analyzed the observations (ie, video recordings). In the fourth step, we compared the CHWs’ behaviors with the counseling ThinkLets to assess their adoption (solution objective 2). Fifth, we applied the critical incidents technique to identify outstandingly effective or ineffective behaviors [78]. Finally, we analyzed the 2 focus group discussions following a theory-driven coding approach based on the empowerment literature [20,25] paired with in-vivo coding for emerging themes. Analyzing the focus group discussions allowed us to understand the insights from the previous steps. The initial codes were *access to information, support, resources,* and *opportunities* (structural empowerment) and *meaning, impact, competence,* and *autonomy* (psychological empowerment).

#### Field Evaluation

We collected a second round of data after the CHWs worked in the field for approximately 12 weeks. The CHWs again completed the same questionnaire on empowerment and system usability. Further, they provided insights into their work through semistructured interviews (individual interviews).

We first analyzed the empowerment questionnaire and SUS score (steps 1 and 2). In step 3, we calculated changes between the initial and second questionnaires in addition to the median and mean. These changes yielded interesting insights into the dynamics of empowerment that we further explored in the interviews (step 5). We analyzed the interviews based on the same coding approach to find explanations for the identified changes. We further analyzed the interviews regarding the continued use or adaptation of the ThinkLets (step 4). Finally, we combined survey data, interviews, and field observations to draw our conclusions. We could generate a rich and multidimensional understanding of Scripted Medicine through careful triangulation. Our abductive approach sensitized us to balance the empirical findings and theoretical grounding in collaboration engineering, algorithmic management, and empowerment. Finally, we formulated design principles as proposed in a study by Gregor et al [79] to synthesize our contribution and inform designers.

### Artifact Description

**Figure 2:**
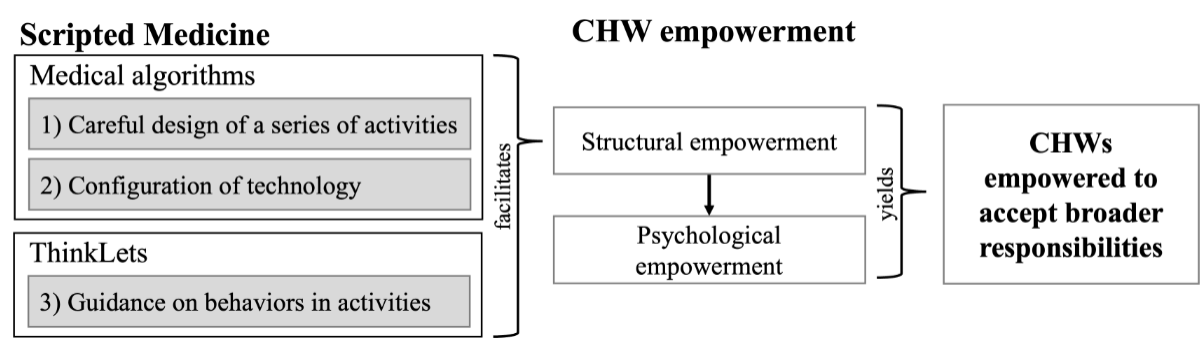
A conceptual model for community health worker (CHW) empowerment with Scripted Medicine based on process restrictions through medical algorithms and ThinkLets.

Scripted Medicine was developed based on the 3 principles of process restriction proposed in collaboration engineering: (1) careful design of a series of activities; (2) configuration of technology to support behaviors within each activity; and (3) guidance on the behaviors (including constraints) performed in each activity [59]. Insights from algorithmic management guided the design of the series of activities and the configuration of technology, while ThinkLets formalize the behaviors in the activities. Scripted Medicine interlinks medical and counseling expertise conveyed by algorithms and ThinkLets to structurally and psychologically empower CHWs. The act and perception of empowerment allow CHWs to accept broader responsibilities in NCD care. Figure 2 depicts this conceptual model for Scripted Medicine.

### Formalizing Clinical Knowledge in ComBaCaL

Medical algorithms formalize the clinical knowledge necessary to offer diagnosis, monitoring, and treatment services for NCDs. These algorithms represent a carefully designed series of activities that follow existing medical guidelines. They constrain CHWs in their actions to ensure the proper application of care procedures for patient safety. These constraints facilitate the expansion of CHWs’ responsibilities and have been shown to empower CHWs [35]. For example, they offer step-by-step guidance to support CHWs in implementing the proper medical procedures in the correct order. Further, mHealth automates documentation, which frees up time for CHWs. Finally, such tools give CHWs access to relevant medical history information collected previously.

**Figure 3:**
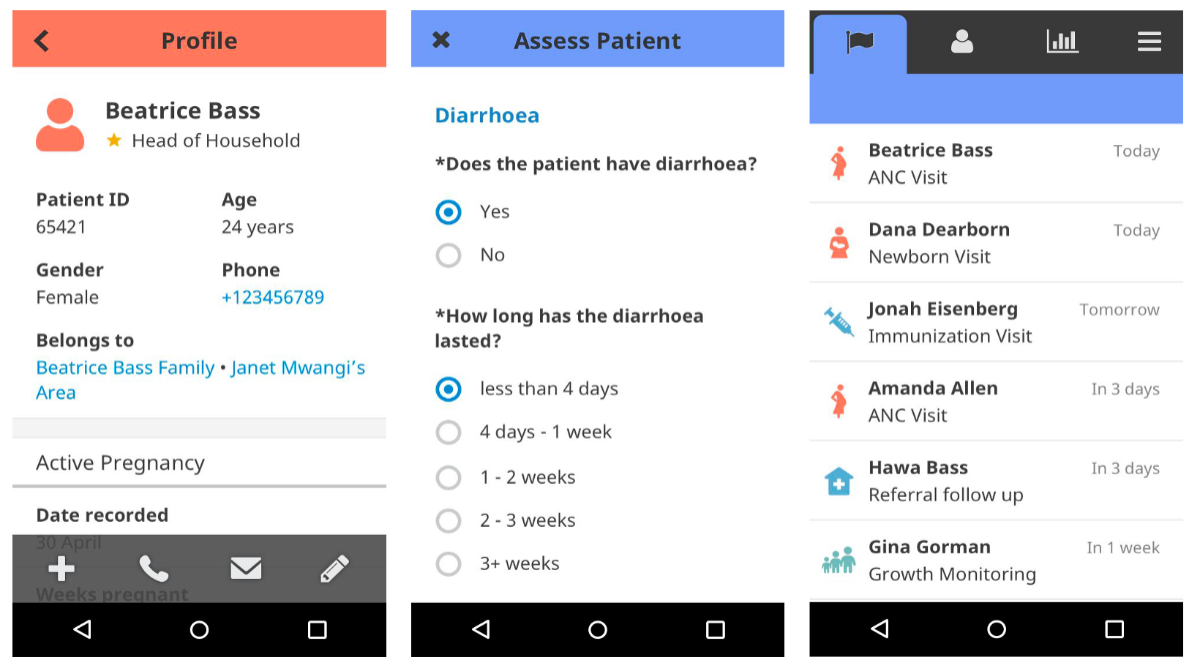
The community health toolkit structure in the tabs: (A) profile; (B) care guides; and (C) tasks. ANC: antenatal care.

The medical algorithms were implemented in CHT and called the ComBaCaL app. The toolkit offers a “shell” with basic functionalities and allows for rapid deployment of medical algorithms. The ComBaCaL app is structured in 3 tabs: tasks, care guides, and profiles (Figure 3 [72]). The medical algorithms influence each tab by defining open tasks and their due date, storing, and displaying information in patient profiles, and providing step-by-step guidance in the care guides. For the study, the medical algorithm covered medical procedures for (1) *patient and household enumeration* (ie, registration and obtaining informed consent); (2) *screening for diabetes*, using capillary blood sugar measurements after prescreening using age and body mass; (3) *diagnosing diabetes*; and (4) *initiating treatment* (ie, medication and lifestyle advice, if applicable). In a diagnosis, the medical algorithm would initiate screening for signs and symptoms of advanced disease and other criteria that would require a referral of the patient to a health facility. In an uncomplicated disease, the algorithm would start a treatment cycle with regular follow-ups to adjust medication until an acceptable blood glucose level is reached. If acceptable blood glucose levels cannot be reached through the treatment provided by the CHW, the algorithm would then suggest referral to the health facility for further care. Guidelines for medical professionals are confined to essential clinical information. More guidance is required in community-based health care as CHWs do not have this formal education. In ComBaCaL, the medical algorithms contained relevant clinical questions, notes on preparations (eg, to ensure that all necessary materials, such as protective gloves are available), information to provide to clients (eg, possible side effects of medication), and alerts, if a client must be sent to a medical professional. However, not all relevant knowledge can be conveyed in algorithms, as they would become cluttered and overly lengthy.

### Formalizing Counseling Knowledge in ComBaCaL

Accordingly, Scripted Medicine formalized counseling knowledge in ThinkLets. Medical scripts (ie, medical algorithms) guided the CHWs through a series of activities. This way, they offered the information basis for the social interactions (eg, a diagnosis a CHW must explain to the patient). ThinkLets formalized counseling expertise to guide CHWs in these social interactions throughout the consultation process. While initially developed to guide behaviors in large group collaboration, ThinkLets are not limited to such collaborations. They have been successfully applied in dyadic collaboration. For example, ThinkLets can empower service providers to use technology to adapt work practices for value cocreation in service encounters [80]. They can also facilitate knowledge transfer between expert advisers and nonexpert clients in burglary prevention [81].

In Scripted Medicine, they contained the counseling knowledge required to interact meaningfully with clients and build quality relationships. Counseling ThinkLets embed the clinical knowledge instantiated in algorithms into social interactions. Such ThinkLets can range from high-level steps to microinteractions. For example, a ThinkLet could provide instructions on approaching new clients and introducing the health program to start counseling. Another ThinkLet on comforting clients could offer CHWs a tool to handle patients’ emotional reactions after a diagnosis.

In ComBaCaL, social interactions along the medical algorithm’s guidance were scripted in 9 ThinkLets. The ThinkLets covered the steps of g*reeting and consent*, e*numeration, screening, confirmation, counseling, referral, medication, dosing, closing with diabetes,* and *closing no diabetes*. These high-level steps are intended as a proof-of-concept that should be broken down into more nuanced ThinkLets with growing experience and insights. Table 2 shows the *greeting and consent* ThinkLet as an example. Italicized texts indicate posttraining adaptations made due to insights from the training. For example, Basotho customs require people entering the property to announce themselves with “Koko.” The ThinkLets indicates the inputs required and expected outputs for the interaction. The setup includes instructions, for example, on how to position oneself or organize the working space. The steps and “say this...” offer guidance on how to structure the interaction. In the subsequent example, the ThinkLet also refers to additional materials to be used (ie, “according to the introduction cheat sheet”). Finally, notes offer insights for CHWs and others into the context and mechanisms of a ThinkLet.

**Table 2:** An example ThinkLet greeting and consent, with posttraining adaptations in italics.

Dimension	Description
Name	Greeting and consent
Inputs	None
Outputs	Signed informed consent form and client who knows the CHW^a^ and ComBaCaL^b^
Setup	*Say loudly “koko” while entering the property and repeat until you can be certain that the owner heard you if they are around*
Steps	Greet clients Briefly present yourself Briefly present ComBaCaL cohort study Wait for permission to take a seat or ask for it (outdoor or indoor) Take tablet and the *introduction cheat sheet*
Say this	*According to the introduction cheat sheet:* present yourself present ComBaCaL explain informed consent form Let all clients sign the informed consent form (including children) *Start creating the household in the app* *Answer all required questions while creating the household* *Submit*
Notes	Basotho houses in rural areas differ from houses in western countries in that you already enter a more private part of the property, even if you have not yet stepped through a front door. Because many houses consist of only 1 room, one already enters the bedroom when entering the house interior. The properties are fenced and when you pass through the fence gate, however, you cannot knock. The observations during the trainings have shown that the CHWs replace the ringing or knocking at the door with a loud “Koko.” This should be loud enough so that it can he heard over the entire property and the owner will be aware of it if they are home.

^a^CHW: community health worker.

^b^ComBaCaL: Community Based Chronic Disease Care Lesotho.

## Results

### Overview

In this section, we report on the evaluation outcomes of Scripted Medicine in the field study in ComBaCaL. We evaluated the attainment of the design goal (ie, empower CHWs to accept broader responsibilities in NCD care) in 2 rounds. First, we examined Scripted Medicine’s effect on CHW empowerment directly after the training. Second, we highlighted changes in the CHWs’ perception after approximately 3 months in the field.

### CHW Empowerment After the Training

The video recordings captured the CHWs’ application of the counseling ThinkLets. While the interactions in practice sessions at the beginning appeared artificial, the CHWs felt noticeably more comfortable interacting with clients in the nearby villages. The analysis showed that most CHWs closely followed the ThinkLets. However, they had already started adjusting the scripts to their liking. For example, instead of enumerating a household with everyone present, they opted to enumerate each member individually and asked others to leave. Later, the CHWs explained that due to the personal questions in the general health questionnaire (eg, HIV status), they wanted to create a more secure environment.

The medical algorithms in the ComBaCaL app scored 74.25 on the SUS. This indicated that the CHWs perceive the medical algorithms and the ComBaCaL app as useful in counseling clients. The analysis of the video recordings supports this indication. Most CHWs navigated the app without difficulties at the end of the practice-oriented training.

The empowerment questionnaire showed that the training empowered the CHWs. On average, the CHWs rated their structural empowerment at 4.5 on a 5-point Likert scale (Table 3; Multimedia Appendix 3 provides further details). In the focus group discussions, many referred to the clear structure provided by the medical algorithms and ThinkLets. One CHW said the following:

I thought it’s going to be hard to go through [all the procedures]. But then, when we proceeded, everything became clearer. The app reminds me, and things come easy.

**Table 3:** Evaluation results of community health worker empowerment on a 5-point Likert scale and System Usability Scale (range 0-100).

Measure and dimension			After training		After field experience		Trend
**Structural empowerment (5-point Likert scale)**							
	Access to opportunities	4.5		4.6		↑	
	Access to information	4.6		4.6		—^a^	
	Access to support	4.3		4		↓	
	Access to resources	4.6		4.3		↓	
**Psychological empowerment (5-point Likert scale)**							
	Meaning	4.9		4.8		↓	
	Competence	4.6		4.5		↓	
	Autonomy	4.1		4.2		↑	
	Impact	3.8		4		↑	
**System Usability Scale (** **range 0-100** **)**							
	Total score	74.25		76.5		↑	

^a^Not available.

The training approach also resonated well with the CHWs. They could make mistakes in a safe environment, where they received feedback from trainers and peers. Further, the Butha Buthe CHWs agreed in the focus group discussion that the last days in the nearby village greatly benefited them in interacting with strangers. Back in their community, their clients mostly will not be strangers but people they already know to a certain degree, which would make the interactions even easier. However, some CHWs, particularly those from Mokhotlong (ie, the more rural district), raised infrastructure concerns such as the lack of electricity to charge their tablets. Consequently, the Mokhotlong CHWs rated their access to resources as 14% lower (4.27 compared to 4.87) and access to support as 17% lower than their Butha Buthe colleagues (3.93 compared to 4.6).

The CHWs also felt empowered as they rated their combined psychological empowerment at 4.35 out of 5. Notably, impact is the only dimension rated <4, at 3.8. Again, the Mokhotlong CHWs rated the perceived impact noticeably lower (26%, 3.33 compared to 4.2). In the focus group discussions, some feared that people might refuse their services due to skepticism, as NCDs “are for the rich people” and would only affect “people who are older.”

Conversely, both groups perceived an exceptional meaning in their future work (4.9 out of 5), which one CHW explained with this:

We don’t want to see an increased number of patients diagnosed with diabetes and hypertension, and also, we don’t want to see people who already have hypertension or diabetes die due to complications of those diseases.

Another CHW highlighted the benefits of the counseling ThinkLets. She described herself as shy, “but this time, I’ve seen that I’m not shy anymore, and I can speak freely to everyone.” The Mokhotlong CHWs all rated meaning with the maximum score and, thereby, slightly higher than the Butha Buthe CHWs. Further, all CHWs felt confident with their new skills and roles. One CHW said the following:

I don’t think there will be challenges. The people in my village are waiting for me.

However, the CHWs also raised some initial concerns. The concerns were primarily related to their acceptance, as community members might not be willing to accept their services. Both groups would prefer a project representative to introduce them to their village to gain initial credibility. Some mentioned that weather conditions influencing their ability to travel were a concern, especially in winter. These concerns often evolved around the loss of control, which could explain the CHWs’ lower perception of autonomy compared to the other dimension (4.1 out of 5 on a 5-point Likert scale).

When asked what they expect upon returning, one CHW emphasizes the collaboration with patients as follows:

We’re expecting to work hand in hand with patients. And being kind to them, to speak to each other. I think the most important thing is the idea of ComBaCaL to come through, and this is going to happen by the way we’ll be working. We are going to be sure that each and everyone will be happy with our services.

Despite raising some concerns, the CHWs felt well-prepared for their new responsibilities when returning to their communities after the training.

### CHW Empowerment After Field Experience

After approximately 12 weeks in the field, the CHWs still felt empowered and ranked the ComBaCaL app slightly higher at 76.5 points on the SUS (Table 3).

Over the 3 months, CHW empowerment showed some minor changes. As the CHWs are distributed across 2 districts in Lesotho, they appeared to perceive less access to support (from 4.3 to 4 out of 5). During the training, they had access to supervisors, experts, and peers to ask questions and receive feedback. After returning to their communities, the CHWs mainly worked alone. In the interview, one CHW proposed adding “a chatting platform, where people are ready to help in case of seeking for help; on which our instructors are able to communicate with us.” Further, they had fewer opportunities to acquire new skills than during the training (from 4.6 to 4.3). Some mentioned in the interview that adjusting from a training setting to working back in their community took some time.

The CHWs perceived the same or even slightly better access to information (from 4.5 to 4.6) and resources (remained at 4.6) compared to the training. One CHW refers to the tablet as the source of information and resources as follows:

It helps a lot since it shows all the activities we were taught in training. It shows which people to attend the following day and how many people I have offered services to. It...helps me to keep track of people who are served and others to be served. If I happen to make a mistake in my activity, it reminds me because it shows that something is wrong.

The CHWs’ psychological empowerment also underwent some changes. Despite their high confidence in their skills, most CHWs report challenges they encountered in their community. Accordingly, perceived meaning and competence slightly decreased. A CHW mentioned convincing young adults as a challenging task as follows:

The main challenge is that the youngest adults refuse to agree when we test for them. But after talking to them about the importance of this, then they agree.

Interestingly, most CHWs reported similar experiences of persuading reluctant clients when asked about their greatest success so far. For example, another CHW said: “what made me happy is the joy of being able to convince a person, or generally making a person have interest in matters of health*.*” They could persuade clients to accept their services despite the initial skepticism. This success might have increased perceived autonomy and impact, as “those one I helped are very impressed since ComBaCaL is taking care of their lives door to door, and they even gained some information on how to take care of themselves*.*” After translating their training into practice, the CHWs could see the impact of their work. This gave them confidence in their capabilities and resulted in more trust from their patients, as one CHW summarized:

Most of my clients see the confidence in me when I walk; when I touch anything. So, when you enter the door of the household, you should look confident and say clear things so clients are able to trust you. [The training] was basic because it helped me overcome my fear. That was my first action when I started; to see how to work with a patient. So, I learned there, and I was able to move forward with the knowledge.

Interestingly, the interviews showed that medical algorithms dominated the perception of structural empowerment. However, psychological empowerment is often attributed to the ThinkLets that embed clinical knowledge in client interactions. As in the example earlier, the CHW refers to “work with a patient” and their confidence in their interactions with the patient. Hence, combining medical and counseling knowledge empowered CHWs to accept their responsibilities in ComBaCaL.

## Discussion

### Principal Findings

This study aimed to design Scripted Medicine, which is an approach to empower CHWs to accept broader responsibilities in NCD care. We grounded the approach in research on collaboration engineering and algorithmic management. Restricting CHWs in the process facilitated the transfer of power (ie, structural empowerment) that led to psychological empowerment. As hypothesized, the empowered CHWs could accept the broadened responsibilities and offer more holistic NCD care to their community. These results allow us to make a 2-fold contribution.

### Scripted Medicine

Scripted Medicine is a novel approach inspired by existing research on collaboration engineering and algorithmic management. It is the product of scripting the necessary medical and counseling expertise. Scripted Medicine combines previously separated knowledge spheres to create significant empowerment potential in community-based health care. Process restrictions empower CHWs by conveying the relevant knowledge required for their work.

Medical algorithms empower CHWs to perform medical procedures [40-42]. Scripted Medicine broadens the perspective on medical algorithms by drawing on algorithmic management literature [43,44,47]. The 5RSM framework provides a nuanced distinction of algorithmic management forms that influence how researchers design and study medical algorithms [47]. For example, our evaluation highlights the necessity for carefully considering algorithmic requiring and recommending. CHWs might feel disempowered when requiring noncritical activities instead of providing certain flexibility in work planning. On the contrary, previous research reports seemingly contradictory observations in CHW empowerment. CHWs perceived algorithmic restricting as empowering instead of a loss of autonomy [54]. This observation aligns with collaboration engineering research [59,60]. Accordingly, Scripted Medicine explains how restricting CHWs in the process (eg, restricting unsafe actions) while allowing enough flexibility for users to adapt to their context is empowering rather than disempowering. It also emphasizes the “well-designed” when instantiating clinical knowledge in mHealth tools. Consequently, we formulate the design principle of scripted medical activities (Textbox 2 [79]).

**The design principle of scripted medical activities following the study by Gregor et al.**

**Title**

- Design principle of scripted medical activities

**Aim, implementer, and user**

- For designers and researchers (implementers) to efficiently and effectively convey medical knowledge (aim; solution objective [SO] medical knowledge) by drawing on the algorithmic recommending, restricting, requiring, rating, rewarding, sanctioning, and monitoring (5RSM) framework to guide community health workers (CHWs; users) through carefully designing a series of medical activities

**Context**

- Community-based health care heavily relies on medical algorithms to enable CHWs to perform medical procedures (eg, diagnostics) safely and independently [40-42]. While requiring CHWs to follow a specific path creates more reliability, it ignores the individual preferences of CHWs and might lead to conflicts with other responsibilities (eg, families). This could result in dropouts [13,14].

**Mechanism**

- Provide medical knowledge to CHWs through scripts based on algorithms (eg, decision trees), allowing them to have agency over noncritical medical activities

**Rationale**

- Collaboration engineering allows nonexperts to perform activities by restricting their process flexibility [59]. While this has an empowering effect, certain forms of algorithmic management (ie, recommending and requiring) can have opposite effects [47]. Our results have shown that requiring a specific order in which tasks are completed feels too restrictive. Allowing CHWs to plan their work independently, within limits, offers them an agency that is perceived as empowering. Accordingly, the 5RSM framework offers more nuanced forms of algorithmic management that acknowledge the need for reliability and safety as well as independence and agency.

Scripted Medicine further argues that formalizing clinical knowledge alone is insufficient for sustainable community-based health care. While medical algorithms provide the foundation to expand CHWs’ responsibilities, they do not adequately address all relevant aspects of empowering CHWs. Medical algorithms cater to 2 out of 3 aspects of process restrictions as follows: (1) careful design of a series of activities and (2) configuration of technology to support behaviors within each activity. However, they lack the final ingredient that translates the clinical knowledge into its application context: (3) guidance on the behaviors performed in each activity to maneuver CHWs and their clients through the counseling process.

Scripted Medicine addresses a significant shortcoming in current research that often assumes that medical algorithms alone empower CHWs. This limited perspective results in unilateral solutions that solely focus on the technical dimension of CHW empowerment [29,30,54]. However, CHW acceptance and overall health outcomes also depend on the quality of the social interactions between CHWs and their clients [52,53]. Yet, counseling knowledge is seldom in scope in community-based health programs. Scripted Medicine extends the existing work on CHW empowerment as it embeds clinical knowledge into the context where it is applied. Our evaluation indicated differences between Mokhotlong and Butha Buthe CHWs. These differences highlight the importance of considering the social context. Both CHW groups received the same scripted expertise and training but showed different perceptions of structural and psychological empowerment. While the survey only offered indications, our observations and interviews allow us to argue that the slightly different contexts between the 2 districts cause the indicated differences. People in Mokhotlong might be more skeptical of Western medicine in general. Hence, CHWs might believe the community will be reluctant to accept their services and perceive less impact of their work. Further, Mokhotlong CHWs raise the issue of lacking access to electricity or cellular signals, while their Butha Buthe colleagues do not. Infrastructure differences also alter empowerment perceptions. Mokhotlong CHWs perceive less access to resources (ie, the tablet could run out of battery) and support (ie, no means to call or text a supervisor). Scripted Medicine obliges designers and researchers to consider the context within which they operate carefully.

Counseling ThinkLets serve multiple purposes in Scripted Medicine. First, ThinkLets were useful training modules for counseling clients with the support of medical algorithms. Trainers can structure training along ThinkLets to maintain a logical order. ThinkLets can first be discussed and demonstrated in the plenum before moving to practice-oriented training sessions. In these sessions, CHWs apply ThinkLets to build expertise in a safe environment and receive feedback from trainers and peers. Second, ThinkLets are individual learning modules beyond official training sessions. CHWs can always revert to them in case they feel insecure. For example, CHWs might not perform specific tasks regularly and want to refresh their memory before counseling clients. Third, ThinkLets are intended to provide a foundation for social interactions in counseling. While the medical algorithm provides continuous constraints for care procedures, ThinkLets should “kickstart” the CHWs’ counseling practices that they adapt over time as their expertise grows. Finally, ThinkLets allow designers and researchers to study the evolving routines of individual CHWs when performing their work. These insights can be formalized and shared with other CHWs to make knowledge accessible within the program.

Scripted Medicine emphasizes the sociotechnical context, which is often lacking in existing literature on technology-supported community–based health care. It allows research to go beyond medical outcomes and consider CHWs’ difficult position at the margin of different communities (eg, their families and the health system), often without fully belonging to any of them [29]. In combination, counseling ThinkLets and medical algorithms facilitate the structural empowerment of CHWs as they gain access to information, resources, support, and opportunities. Scripted Medicine postulates a more comprehensive view of structural empowerment, as optimizing one aspect might not cater to all dimensions. For example, medical algorithms might reduce or even prevent access to opportunities. CHWs cannot deviate from the algorithm’s predetermined workflow to ensure patient safety. On the other hand, counseling ThinkLets open plenty of opportunities for CHWs to develop their personal way of interacting with clients. They empower CHWs to transfer medical procedures from isolation into the social interactions of health services. On the basis of these insights, we formulate the design principle of scripted counseling (Textbox 3 [79]).

**The design principle of scripted counseling following the study by Gregor et al.**

**Title**

- Design principle of scripted counseling

**Aim, implementer, and user**

- For designers and researchers (implementers) to efficiently and effectively convey counseling knowledge (aim; solution objective [SO] counseling knowledge) to support community health workers (CHWs; users) in the creation of their own, culturally adapted counseling practices

**Context**

- Digital health tools for community-based health care primarily convey medical knowledge to CHWs [29,30,54]. While medical knowledge is crucial, health care is always an interaction between people. Accordingly, existing research does not support CHWs enough to transfer this medical knowledge into action.

**Mechanism**

- Provide counseling knowledge to CHWs through ThinkLets (ie, communication scripts), allowing them to scaffold their advice and to develop their counseling practices in accordance with their preferences and the cultural context

**Rationale**

- CHW acceptance and health outcomes significantly rely on social interactions [52,53]. Counseling ThinkLets require designers to consider the context for which they design and allow CHWs to develop their own practices that suit their preferences and strengths. Further, it allows tailoring to nuances within specific cultural contexts. Our findings indicated differences between the CHWs from the more urban Butha Buthe and the rural Mokhotlong.

Scripted Medicine is more than stringing together medical and counseling scripts. These scripts are interdependent. Explicating medical knowledge in the form of guidelines has long been established. Counseling knowledge, as established in the introduction, is often implicit and gained with years of training and experience. Accordingly, guidelines for medical professionals focus on essential clinical information. They assume medical professionals possess the counseling experience to translate clinical information into the consultation context.

The literature often neglects that CHWs require more guidance than medical professionals. Scripted Medicine is an approach to formalizing both areas of expertise, as clinical knowledge alone is insufficient. For example, CHWs can counsel people if they can rely on proper medical guidance and access relevant clinical information about patients and medical procedures. However, access to that data and being able to implement the medical procedures depends on a good working relationship with patients. Establishing and maintaining such a relationship requires guidance on counseling practices.

Scripted Medicine considers the symbiosis of medical and counseling knowledge and their interdependence. Accordingly, it is more than merely a combination of clinical and counseling guidance. For example, medical algorithms might include different media types for CHWs to educate patients about their condition after a diagnosis. Further, ThinkLets must consider the process restrictions implemented in algorithms to avoid unnatural interactions. This combination of formalized knowledge in Scripted Medicine is the foundation for psychological empowerment. While clinical knowledge itself might enable CHWs to conduct medical procedures, CHWs perceive the embedding in social interactions as empowering. It allows CHWs to see the meaning in their work, feel competent in counseling clients, autonomously plan their work, and observe their work’s impact on their community. Thus, we formulate the design principle of Scripted Medicine (Textbox 4 [79]).

**The design principle of Scripted Medicine following the study by Gregor et al.**

**Title**

- Design principle of scripted medicine

**Aim, implementer, and user**

- For designers and researchers (implementers) to meaningfully intertwine formalized medical and counseling knowledge (aim) to empower community health workers (CHWs; users) to accept broader responsibilities in noncommunicable disease care

**Context**

- On one side, CHWs depend on medical algorithms to offer health services. On other side, medical algorithms depend on the data inserted by CHWs during consultations. Thus, CHWs require a tool that carefully aligns the designed medical activities with counseling practices to carry out more complex tasks successfully.

**Mechanism**

- Integrate formalized medical and counseling knowledge visually and algorithmically to empower CHWs

**Rationale**

- Collaboration engineering is an approach that carefully considers the effects of restricting and guiding users through activities and interactions [57,59]. IT-supported community–based health care is a sociotechnical system in which people, tasks, systems, and organizational structures (communities) are inherently interdependent [54]. Thus, CHWs require access to information, resources, support, and opportunities that consider the social and technical dimensions of community-based health care.

### Becoming Agents of Social Change

Existing research provides in-depth insights into the design of mHealth to capacitate CHWs to perform actions they could not perform otherwise, such as diabetes screening [5,34,37,41]. While this work is fundamental for expanding CHWs’ responsibilities, it fosters a unilateral focus on conveying medical knowledge through mHealth. It has been argued that most studies view CHWs as sensors of the health system, leading to a reductionist perspective on community-based health care [54]. In other words, existing research almost exclusively focuses on the technical domain in a sociotechnical system and omits the social dimension. This situation was mentioned in a critique by Engel [82] on biomedical medicine as follows:

The crippling flaw of the [biomedical] model is that it does not include the patient and [their] attributes as a person, as a human being. Yet, in the everyday work of the physician, the prime object of study is a person.

Similarly, existing research does not consider community-based health care as a collaborative process between CHWs and their clients. However, multiple studies highlight the importance of social interactions in community-based health care for patient experience and treatment adherence [52,53]. Scripted Medicine offers a lens that emphasizes the collaborative nature of community-based health care. While the immense benefits of community-based health care are undoubted, we criticize the often reductionist consideration of health outcomes only. mHealth might increase CHW performance and retention or capacitate them to perform more complex tasks guided by medical algorithms [36]. However, they do not necessarily address the root causes of low performance and high attrition (eg, lack of organizational support and appreciation [9,14]). Accordingly, mHealth could even worsen the situation for CHWs. Their responsibilities continuously broaden and become more complex, while their work conditions do not necessarily improve at the same rate. From an empowerment perspective, current research focuses on shifting responsibilities and often forgets shifting power. This could lead to CHWs feeling more disempowered and increase instead of decrease attrition rates over time.

Scripted Medicine aims to address such structural issues. It accentuates the interdependence between the 2 dimensions of health care: the medical and outcome-oriented procedure (health) and the interpersonal, collaborative character (care). For example, existing reports on health outcomes of community-based health initiatives are limited to people willing to accept CHWs’ services. Reluctant people do not benefit from improved access to health care and, consequently, are usually not considered in such studies. Our evaluation indicates that Scripted Medicine might empower CHWs to better persuade this population to accept their services. By considering both dimensions of health care, we believe CHWs become better service providers and become the “agents of social change, beyond their role as links between the community and the health system.” [50]

### Conclusions and Limitations

Task shifting to CHWs is a promising remedy to the imminent shortage of medical professionals in the Global South [4,5]. However, community-based health programs often face performance variability and high attrition rates [9-13]. As a result, task-shifting efforts might be built on unstable foundations if the root causes of the existing challenges are not resolved.

Scripted Medicine provides a more comprehensive perspective to empower CHWs to accept broader responsibilities in NCD care. Scripted Medicine “scripts” clinical and counseling knowledge that promises to quickly empower CHWs to accept broader responsibilities and become active members of the health system. It draws on research on collaboration engineering and algorithmic management to restrict CHWs in the counseling process in a way that empowers them to offer more complex health services, such as first-line treatment for uncomplicated type 2 diabetes and hypertension.

Scripted Medicine positions CHWs as active members and not sensors of the health system. Scripting medical and counseling knowledge transfers the power necessary to offer high-quality health services to CHWs. In turn, CHWs feel empowered to accept new responsibilities and, thereby, increase the accessibility of health care. Scripted Medicine highlights the need for a deeper understanding of the local context and the design of contextually adapted community-based health programs to support CHWs in their work.

However, this study does not come without limitations. Scripted Medicine broadens the unilateral focus on the technical dimension by emphasizing social interactions. However, more research is needed to study other potential dimensions influencing CHW empowerment. Empowerment is a dynamic concept that requires a longitudinal assessment of changing CHW demands [35]. Finally, the rich dataset allowed us to triangulate Scripted Medicine from empirical data. Future work is needed to validate these empirical observations at a larger scale.

## Appendix

Multimedia Appendix 1 The empowerment questionnaire as administered in the study with Sesotho translation, adapted from a study by Spence Laschinger et al.

Multimedia Appendix 2 The System Usability Scale as administered in the study with Sesotho translation, adapted from a study by Bangor et al.

Multimedia Appendix 3 Survey results for the System Usability Scale and structural and psychological empowerment per community health worker.

## Abbreviations

5RSM: algorithmic recommending, restricting, requiring, rating, rewarding, sanctioning, and monitoring
CHT: community health toolkit
CHW: community health worker
ComBaCaL: Community Based Chronic Disease Care Lesotho
GSS: group support system
mHealth: mobile health
NCD: noncommunicable disease
SUS: System Usability Scale
