# Supplementary material for: Empowering Community Health Workers With Scripted Medicine: Design Science Research Study
Source: JMIR Hum Factors. 2025 Apr 23;12:e57545. doi: 10.2196/57545 (PMC12059493; doi:10.2196/57545)
Supplement: Multimedia Appendix 1 [file humanfactors_v12i1e57545_app1.pdf]

Empowerment

Meaning

The work I do is very important to me

Mosebetsi oaka o bohlokoa

Strongly Disagree | Disagree | Neutral | Agree | Strongly Agree

My job activities are personally meaningful to me

Mosebetsi oaka ona le boleng ho nna

Strongly Disagree | Disagree | Neutral | Agree | Strongly Agree

The work I do is meaningful to me

Mosebesti eke o etsang o bohlokoa ho nna

Strongly Disagree | Disagree | Neutral | Agree | Strongly Agree

Competence

I am confident about my ability to do my job

Ke kholisehile ka bokhoni ba ka ba ho etsa mosebetsi oa ka

Strongly Disagree | Disagree | Neutral | Agree | Strongly Agree

I am self-assured about my capabilities to perform my work activities

Ke kholisehile ka bokhoni ba ka ba ho etsa mosebetsi oa ka

Strongly Disagree | Disagree | Neutral | Agree | Strongly Agree

I have mastered the skills necessary for my job

Ke ithutile tsebo e hlokahalang bakeng sa mosebetsi oa ka

Strongly Disagree | Disagree | Neutral | Agree | Strongly Agree

Self-Determination

I have significant autonomy in determining how I do my job

Ke na le boikemelo bo boholo ba ho etsa qeto ea hore na ke etsa mosebetsi oa ka joang

Strongly Disagree | Disagree | Neutral | Agree | Strongly Agree

I can decide on my own how to go about doing my work

Ke khona ho iketsetsa qeto ea hore na ke tla etsa mosebetsi oa ka joang

Strongly Disagree | Disagree | Neutral | Agree | Strongly Agree

I have considerable opportunity for independence and freedom in how I do my job

Ke na le monyetla o moholo oa boipuso le tokoloho tseleng eo ke etsang mosebetsi oa ka ka eona

Strongly Disagree | Disagree | Neutral | Agree | Strongly Agree

Impact

My impact on what happens in my community is large

Tšusumetso ea ka ho se etsahalang sebakeng sa heso e kholo

Strongly Disagree | Disagree | Neutral | Agree | Strongly Agree

I have a great deal of control over what happens in my community

Ke na le matla a ho laola se etsahalang sebakeng sa heso

Strongly Disagree | Disagree | Neutral | Agree | Strongly Agree

I have significant influence over what happens in my community

Ke na le tšusumetso e kholo ho se etsahalang sebakeng sa heso

Strongly Disagree | Disagree | Neutral | Agree | Strongly Agree

How much of each kind of opportunity do you have in your job as CCW?

Challenging Work

mosebetsi o nang le phephetso

None | . | Some | . | A Lot

The chance to gain new skills and knowledge on the job

Monyetla oa ho fumana tsebo e ncha le thuto ea hao mosebetsing

None | . | Some | . | A Lot

Tasks that use all of your own skills and knowledge

Mesebetsi e sebelisang tsebo le thuto ea hau kaofela

None | . | Some | . | A Lot

How much access to support do you have in your job as CCW?

The current state of the project

Boemo ba hona joale ba sechaba sa heso

None . Some . A Lot

The values of project steering committee

Boleng ba ComBaCaL

None . Some . A Lot

The goals of project steering committee

Sepheo sa ComBaCaL

None . Some . A Lot

How much access to support do you have in your job as CCW?

Specific information about things you do well

Lintlha tse khethehileng mabapi le lintho tseo u li etsang hantle

None . Some . A Lot

Specific comments about things you could improve

Maikutlo a khethehileng mabapi le lintho tseo u ka li ntlafatsang

None . Some . A Lot

Helpful hints or problem solving advice

Litlhahiso tse thusang kapa keletso ea ho rarolla bothata

None . Some . A Lot

How much access to resources do you have in your job as CCW?

Time available to do necessary paperwork

Nako e teng ea ho etsa litokomane tse hlokahalang

None . Some . A Lot

Time available to accomplish job requirements

Nako e teng ho fihlela litlhoko tsa mosebetsi

None . Some . A Lot

Acquiring temporary help when needed

Ho fumana thuso ea nakoana ha ho hlokahala

None . Some . A Lot

In my work job as CCW:

the rewards for innovation on the job are

meputso ea boiqapelo mosebetsing ke

None . Some . A Lot

the amount of flexibility in my job is

boholo ba ho feto-fetoha ha mosebetsi oa ka ke

None . Some . A Lot

the amount of visibility of my work-related activities within the project is

palo ea ponahalo ea mesebetsi ea ka e amanang le mosebetsi ka hare ho ComBaCaL ke

None . Some . A Lot

How much opportunity do you have for these activities in your present job:

Collaborating on patient care with physicians/nurses

Ho sebelisana 'moho mabapi le tlhokomelo ea bakuli le lingaka le baoki

None . Some . A Lot

Being sought out by peers for help with problems

Ho batloa ke lithaka ho fumana thuso ka mathata

None . Some . A Lot

Being sought out by supervisors for help with problems

Ho batloa ke baokameli bakeng sa thuso ka mathata

None . Some . A Lot

Seeking out ideas from professionals other than supervisors, e.g. physicians, pharmacists, dieticians

Ho batla maikutlo ho tsoa ho litsebi tse ling ntle le batsamaisi, mohlala. lingaka, litsebi tsa meriana, litsebi tsa lijo

None . Some . A Lot

How much of each kind of opportunity do you have in your present job?

Overall, my current work environment empowers me to accomplish my work in an effective manner

Ka kakaretso, sebaka sa ka sa mosebetsi sa hona joale se nthusa hore ke phethe mosebetsi oa ka ka tsela e atlehileng

None . Some . A Lot

Overall, I consider my workplace to be an empowering environment

Ka kakaretso, ke nka sebaka sa ka sa mosebetsi e le sebaka se matlafatsang

None . Some . A Lot
