# Supplementary material for: Empowering Community Health Workers With Scripted Medicine: Design Science Research Study
Source: JMIR Hum Factors. 2025 Apr 23;12:e57545. doi: 10.2196/57545 (PMC12059493; doi:10.2196/57545)
Supplement: Multimedia Appendix 2 [file humanfactors_v12i1e57545_app2.pdf]

Usability

I think that I would like to use CHT app on a regular basis.

Ke nahana ke tla thabela ho sebelisa marang-rang a CHT khafetsa.

Strongly Disagree | Disagree | Neutral | Agree | Strongly Agree

I found the CHT app unnecessarily complex.

Ke fumana hore marang-rang a CHT asa sebelisehe ha bonolo.

Strongly Disagree | Disagree | Neutral | Agree | Strongly Agree

I think the CHT app was easy to use.

Ke nahana marang-rang a CHT a sebeliseha ha bonolo.

Strongly Disagree | Disagree | Neutral | Agree | Strongly Agree

I think I would need the assistance of a knowledgeable person to use the CHT app.

Ke nahana ke hloka thuso ea motho a nang le litsebo ho sebelisa marang rang a CHT.

Strongly Disagree | Disagree | Neutral | Agree | Strongly Agree

I thought the various functions of the CHT app were well integrated.

Ke nahana lintlha tsa marang-rang li hokahanya hantle.

Strongly Disagree | Disagree | Neutral | Agree | Strongly Agree

I think the CHT app is too inconsistent.

Ke nahana marang-rang a CHT ha tsitsa.

Strongly Disagree | Disagree | Neutral | Agree | Strongly Agree

I believe that most people would learn to deal with the CHT app very quickly.

Ke nahana batho batla ithuta kapele ho sebetsa le ho sebelisa marang-rang a CHT.

Strongly Disagree | Disagree | Neutral | Agree | Strongly Agree

I found the CHT app very cumbersome to use.

Ke fumana marang-rang a CHT ale boima hoa sebelisa.

Strongly Disagree | Disagree | Neutral | Agree | Strongly Agree

I felt very safe using the CHT app.

Ke utloa ke tsireletsehile ho sebelisa marang-rang a CHT.

Strongly Disagree | Disagree | Neutral | Agree | Strongly Agree

I had to learn many things before I could work with the CHT app.

Ke ile ka tlameha ho ithuta ntho tse ngata pele nka sebelisa marang-rang a CHT.

Strongly Disagree | Disagree | Neutral | Agree | Strongly Agree
